# Pathological Characteristics, Prognostic Determinants and the Outcome of Patients Diagnosed with Colorectal Adenocarcinoma at the University Teaching Hospital of Kigali

**DOI:** 10.1155/2022/6608870

**Published:** 2022-09-20

**Authors:** Delphine Uwamariya, Déogratias Ruhangaza, Belson Rugwizangoga

**Affiliations:** ^1^Department of Pathology, School of Medicine and Pharmacy, University of Rwanda, Kigali, Rwanda; ^2^Department of Pathology, Butaro Cancer Center of Excellence, Burera, Rwanda; ^3^Department of Pathology, University Teaching Hospital of Kigali, Kigali, Rwanda

## Abstract

Worldwide, colorectal cancer (CRC) is the second most diagnosed cancer in female and the third in men, arising from the epithelium of the colorectum. It is known that colorectal cancer is common in developed countries than in developing countries which may be due to inaccurate data on the existence of the disease in that region combined with embracing western lifestyle expressed by the current trend of changes in cultural, social, and lifestyle practices playing a major part in the etiology of CRC. The aim of this study was to document epidemiological, pathological characteristics, and prognostics determinants of patients diagnosed with CRC in Rwanda. The data from patients' files and reviewed glass slides for 101 cases all from Kigali University Teaching Hospital (CHUK) were statistically analyzed and patient characteristics were described as mean and frequency accordingly. Comparisons were performed using chi square tests, Fisher's exact test and odds ratio with 95% confidence interval (CI). Survival curves were plotted using the Kaplan–Meier method, and log-rank test was used to assess the statistical differences in the observed survival curves by each categorical variable. A *P* value < 0.05 was considered statistically significant. Statistical analyses were performed using Statistical Product and Service Solutions (SPSS), GraphPad Prism, and MedCalc, accordingly. Mean age of the participants was 54.26 years, the main symptom was rectal bleeding (46.5%), rectal adenocarcinoma NOS represented 40.6%, conventional adenocarcinoma was 60.4%, most tumors were of Grade II (54.5%), most common stage was pT3N0 (20.8%), resection margins were free at 71.3%, lympho-vascular invasion was 49.5% of cases, a high immune response was in 71.3% of cases and of 101cases, and 55.4% were still alive at the end of the data collection, with 29.3% of patients have overall survival of 5 years. Prognostic determinants also affect the outcome in this study and overall survival period was 3 years for CRC diagnosed in Rwanda.

## 1. Introduction

In Africa, CRC is at 6th position after breast, cervix, stomach, prostate, and hematological malignancies [1]. There is a perception that colorectal cancer is more frequent in developed countries due to their western diet and may be an uncommon disease in sub-Saharan Africa [1]. However, this may be related to poor epidemiological data on cancer in general in that region. African countries are embracing the civilization which may play a big role in the increase of colorectal cancer incidence and also raise the burden of the disease in the region [2].

Colorectal carcinoma occupies 90–95% of all colorectal malignancies, and more than 90% of colorectal carcinomas are adenocarcinomas originating from epithelial cells of the colorectal mucosa [3, 4]. Other rare types of colorectal carcinomas include neuroendocrine, squamous cell, adenosquamous, spindle cell, and undifferentiated carcinomas. The present study focused on adenocarcinoma category in which the main subtype is conventional adenocarcinoma and is characterized by the glandular formation which also plays the major role on the concept of histologic tumor grading.

Data related to colorectal adenocarcinomas in Rwanda are scanty except few epidemiological information from our national cancer registry. The purpose of this study is to provide basic clinicopathological information, available prognostic determinants, and the outcome of patients diagnosed with colorectal adenocarcinomas in Rwanda which can help to develop strong measures in terms of prevention and control of the disease in the country.

## 2. Materials and Methods

### 2.1. Patient Population

This was a retrospective descriptive observational study extending from 1st January 2014 to 31st December 2020 targeting all cases with confirmed colorectal adenocarcinoma diagnosed at the largest hospital in Rwanda which is University Teaching Hospital of Kigali (CHUK) in the histopathology laboratory unit. Biopsies and files whose patients underwent surgical resection were included in the study, those for nonoperated patients were excluded as they could not provide most pathology/prognostic data to be collected.

This study was done in order to determine the pathological characteristics, prognostic determinants, and the outcome of patients diagnosed with colorectal adenocarcinoma at CHUK, where glass slides were reexamined by double blinded researchers with a third opinion where it is necessary for diagnosis confirmation and other histological prognostic determinants including immune response, lympho-vascular and perineural invasion, tumor growth pattern, and margin status. From patients' files, epidemiologic data, disease characteristics, and overall survivor were checked during the aforementioned period. The study design was very important because it covered a wider period of seven years and it was approved by the hospital's ethics committee and Institution Review Board of University of Rwanda.

### 2.2. Statistical Analysis

Patients' characteristics are described as mean for quantitative variables and as frequency (percentage) for categorical variables. Comparisons were performed using chi square tests, Fisher's exact test, and odds ratio with 95% confidence interval (CI) as appropriate. We measured survival as the time from the date of diagnosis until the date of death, regardless of the cause, or loss to follow-up, or censoring on 31st December 2020. Survival curves were plotted using the Kaplan–Meier method. The log-rank test was used to assess the statistical differences in the observed survival curves by each categorical variable, and logistic regression analysis was used to predict the impact of each of the factors that showed an association with the survival on univariate analysis. For all analyses, a *P* value < 0.05 was considered statistically significant. Statistical analyses were performed using Statistical Product and Service Solutions (SPSS) version 26 (IBM Corporation, New York 10504–1722, USA), GraphPad Prism (GraphPad Software, Inc., CA 92037 USA) version 9, and MedCalc (MedCalc Software, Mariakerke, Belgium) v.10.2.0.0, accordingly.

### 2.3. Pathological Characteristics Definitions

For the grading we used the WHO of digestive tumors 5th edition grading system which grades CRC according to glandular differentiation, and we used combined clinical and pathological staging according to the WHO of digestive tumors 5th edition [5].

**Figure 1:**
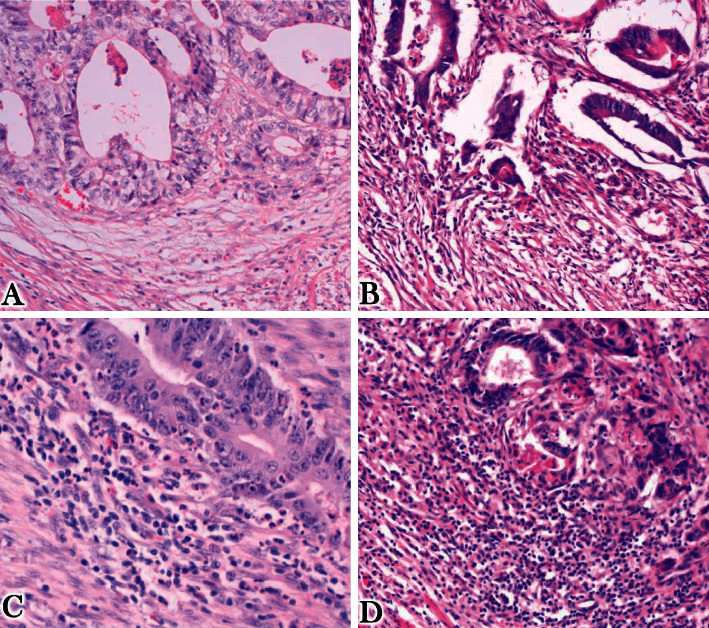
Microphotographs illustrating the inflammatory response:(a) score 0, (b) score 1, (c) score 2, and (d) score 3. For interpretation, scores 0 and score 1 are considered low immune reaction, while score 2 and score 3 denote a high immune reaction.

For an inflammatory reaction, we graded it according to the Klintrup–Mäkinen (KM) system where inflammatory cell infiltrates were evaluated at the deepest large tumor nodule using a 4-point scale, patients were subsequently classified as low grade if they have 0 or 1 and high grade if they have 2 or 3 points ([6] Figure 1).

## 3. Results

### 3.1. Patients' Demographic and Clinico-Pathological Information

During the study period a total number of 101 colorectal resection specimens were collected for the study, patients' demographic and clinical information were retrieved from the archive of the University Teaching Hospital of Kigali (CHUK). The age of the participants ranges between 17 and 89 years with a mean age of 54.26. Females were 53 (52.5%), males were 48 (47.5%), and the predominating age group were 51–75 which comprises 54 patients (Table 1). Many of the attendees were coming from Kigali city with a number of 33 (32.7%) and West and North provinces were the residences providing a small number of patients, each of them provided 13 (12.9%) cases. The main symptom was rectal bleeding with a frequency of 47 (46.5%) and the duration of symptoms was less than 6 months with a frequency of 53 (52.5%). A high number of tumors was found to arise in the rectum 41 (40.6%). The majority of CRC diagnosed at CHUK showed infiltrating pattern with a frequency of 69 (68.3%) and the conventional adenocarcinoma was the main histologic type, it accounted for 61 (60.4%) cases (Table 2).

### 3.2. Pathologic Prognostic Determinants of Colorectal Carcinomas

**Figure 2:**
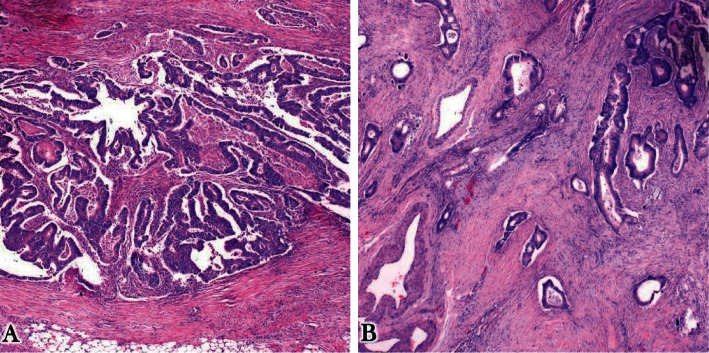
Microphotographs illustrating tumor borders: (a) expansile tumor border and (b) infiltrating tumor border.

Many tumors were of Grade II with a frequency of 55 (54.5%) and the most common staging group was stage III with a frequency of 22 (21.8%) however most of the patients 31(30.7%) were not staged due to lack of their metastasis status (Table 3). A total number of 72 (71.3%) specimen were having radial margins which are free from the tumor and no case was found with proximal or distal margins positive. Most of the tumor borders were irregular infiltrating in 56 (55.4%) (Figure 2(b)); there was no lymph vascular invasion in 52 (51.5%) specimens and no perineural invasion in 72 (71.3%) specimens. There was a high immune response in 72 (71.3%) cases (Figures 1(c) and 1)d).

### 3.3. Association between Different Variables

**Figure 3:**
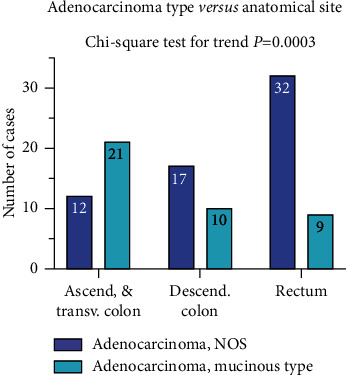
Correlation between anatomical location of the tumor and histologic type NOS (not otherwise specified).

There is a strong correlation between the sex and age groups where it shows that being a male by gender prone someone to develop CRC at a younger age 2.7 times more than a female and this is statistically significant with an OR = 2.7 (95%CI = 1.2–6.2) and *P* value = 0.01 (Table 4). All prognostic determinants studied are associated with tumor grading and it is statistically significant for all parameters (Table 5). There is a strong association between anatomical location and histologic type, where it is shown that conventional adenocarcinomas are mainly found in rectum, and mucinous adenocarcinomas are mainly found in the ascending colon (Figure 3). This is statistically significant with a *P* value = 0.003 (chi-square test for trend).

### 3.4. The Outcome of Patients Diagnosed with Colorectal Carcinoma Operated on at CHUK

**Figure 4:**
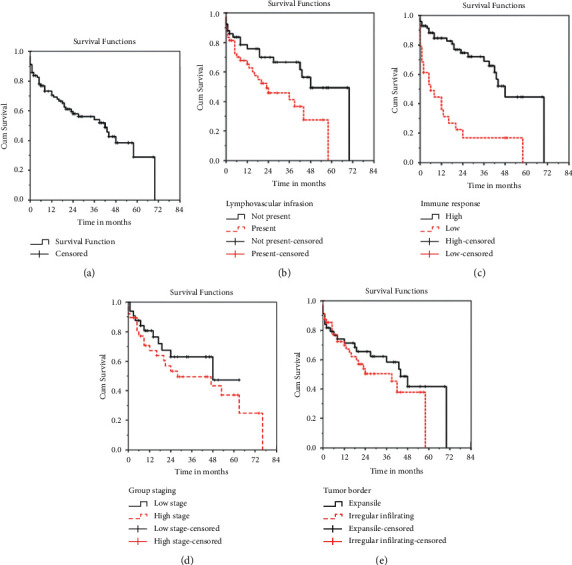
Kaplan–Meier survival curves: (a) Kaplan–Meier overall survival curve, (b) Kaplan–Meier survival according to lympho-vascular invasion: *log-rank test* and *P* value *=* *0.018*, (c) Kaplan–Meier survival according to the immune response status: *log-rank test* and *P* value *=* *0.001*, (d) Kaplan–Meier survival according to the stage groups: *log-rank test* and *P* value *=* *0.227*, and (e) Kaplan–Meier survival according to the tumor border: *log-rank test* and *P* value *=* *0.321*.

Of 101 patients diagnosed with CRC from the year 2014 to 2020, 56 (55.4%) were still alive up to the time of this study (Table 6) and at least 73.1% of the patients diagnosed with colorectal carcinoma lived for 1 year after the diagnosis (Figure 4(a)). Only 29.3% could live between 5 and 6 years.

The prognostic factors studied affect the outcome of patients diagnosed with colorectal adenocarcinoma, some of which are statistically significant like lympho-vascular invasion and immune response (Figures 4(b)–4(c)) where patients whose biopsies were not having lympho-vascular invasion also have an improved survival with a mean of 50 months period, patients who have a high inflammatory response also live longer with a mean of 51 months period and these are statistically significant with a *P* value = 0.018 and 0.001, however only the immune response was independently associated with survival on logistic regression analysis (*P* value = 0.001, and OR = 5.888, CI =(2.137–16.225)) (Table 7) and other prognostic determinants are not statistically significant like the tumor border and stage group (Figures 4(d)–4(e)). Age group, sex, tumor grade, margin status, and perineural invasion also affect the outcome of CRC patients; data are not shown.

## 4. Discussions

The aim of this study was to describe demographic and pathology characteristics, prognostic determinants, and the outcome of patients diagnosed with colorectal adenocarcinoma at the University Teaching Hospital of Kigali (CHUK). In this study, the age of the participants at the presentation ranges between 17 and 89 years with a mean age of 54.26, in general, patients come for diagnosis when they are in their 50 s. The finding about the age range is comparable to the one found in the study done in Zambia where the range was 11 to 82 years of age. In the same study, the mean age was 48.6, a finding which is lower than the one presented in the current study [7]. Females were 53 (52.5%) and males were 48 (47.5%), this means that women tend to seek health care than men [8], it can also be due a natural phenomenon where female are much more presented in the general population than men. This finding is similar to the one found in the study which was conducted in Uganda where the percentage of female was 53.4% [9].

With five regions of Rwanda represented, most of the specimens were provided by patients coming from the capital city of Rwanda, Kigali, with 32.7% and the southern region accounting for 24.8%. This is no surprising since many of the health facilities where cancer diagnosis is done, are in the capital, and people who live in the capital city in one way or the other can afford the diagnosis or are more educated with a higher awareness about cancer, which makes them to consult the doctor. The southern region is also bordering the Kigali city. This finding is similar to others conducted in Tanzania and Zambia, whereby the majority of patients would also come from the respective capital cities [2, 7]. The main symptom was rectal bleeding with a frequency of 46.5%, this was similar to the study published in Zambia [7]. A high number of tumors was found to arise in the rectum with 40.6% of cases which is also similar to the one published in Zambia by Akwi Wasi Asombang1 et al. [7].

The majority of CRC showed gross appearance of the infiltrating pattern with an occurrence of 68.3%, this is similar to what have been found within another study conducted in Europe/Greece by Papagiorgis et al. where infiltrative growth pattern was seen in 38 –32% of the patients in contrast to patients (23 = 20%) with exophytic or polypoid growth pattern [10]. Conventional adenocarcinoma or adenocarcinoma NOS was the main histologic type, it accounted for 60 –60.4% of cases. This was also similar to the one from Zambia [7].

The present study reveals that many tumors were of Grade II accounting for 54.5% and the most common stage group was stage III with 21.8%. With regard to the stage, previous studies have reported similar findings, in the study of Saidi et al., 2011; stage III was most prevalent with 40.1% [11], while a percentage of 43.8% was also reported for stage III elsewhere in Korea [12]. In their study, Papagiorgis et al. also found Grade II to be the commonest grade [10]. Most tumors were also found to be of moderate tumor differentiation in another study done in China with 76.5% of cases. In the same study, tumors with T3 were 40%, whereas tumors with NO were 48% [13].

The incidence of the lymph vascular, margins status, and perineural invasion were 48.5%, 28.7%, and 28.7%, respectively. In a retrospective review which was performed in the USA on the National Cancer Database (NCDB), 2004–2011, positive margins were 39.7% [14]. In their study, Yahyazadeh et al. found that lymph vascular invasion was present in 16.4%, whereas perineural invasion was 30.7%, the study was conducted in Iran [15]. The results about perineural invasion and margins status from those two studies are comparable with the results of the present study. Considering lymphocytic infiltration on H and E staining, there was a high immune response in 71.3% cases, though IHC (CD8 and CD3) were not performed for the characterization of those lymphocytes, but previous research studies have indicated that local lymphocytosis is associated with a favorable prognosis as it has been demonstrated by Klintrup and Mäkinen [6, 15–17]. Also, it is known that cytotoxic T lymphocyte antigen-4 expressed in tumoral infiltrating lymphocytes and CRC cells is an inhibitory immune check point that can interfere with antitumoral drugs and cause poor prognosis with metastasis of the disease in some cases [18]. Further research studies are recommended for a more precision medicine.

The present study demonstrates a positive correlation between the male gender and below 50 years age group where being male will expose you to develop colorectal carcinoma in the younger age than females in the same age group and this is statistically significant with a *P* value = 0.01 and OR:2.7 (95%CI = 1.2–6.2); this was also found by Katalambula et al. [l] in their study done in Tanzanian hospitals [2].

The present study shows that the frequency of conventional adenocarcinoma was higher in the rectum followed by the descending colon and finally the ascending colon, whereas in contrast, the frequency of mucinous adenocarcinoma was higher in the ascending colon followed by the descending colon and finally the rectum (Chi-square test for trend p = 0.003). This implies that in this cohort, rectal cancer tends to be conventional adenocarcinoma, while ascending colon cancer tends to be mucinous adenocarcinoma. It is similar to what have been described by Michelle McCabe et al. [l] in their study where they found conventional adenocarcinoma to arise mainly in the rectum [19].

While it is early to ascertain with confidence the survival outcome, 55.4% of patients diagnosed with CRC at CHUK are still alive. For all patients who survived, the average survival period is 46 months (3 years).

Overall, the results of the present study show no statistically significant (*P* value = 0.253) relationship between the histologic staging group and the CRC outcome where lower stage group I and II live longer with a mean of 51 months period, a finding which is similar to the one found by Bardakhchyan S et al. in their 9 years retrospective study conducted in Armenian oncology center, where they found a 3- and 5-year OS rates were 62.9% and 51.8% for all stages combined and 79.7% and 68.5% for stages I–II, 62.5% and 48.4% for stage III, and 24.4% and 17% for stage IV respectively [20]. The results of the study show that Grade I survived longer than others with a mean of 47 months period. Though, not statistically significant (*P*value = 0.255), it appears that patients with low grades tend to survive longer than high grades. This is similar to what was demonstrated by Bardakhchyan et al. 2020; where tumor grade was considered as the most influential prognostic factor together with the tumor stage [20].

Though it is not statistically significant, the current study shows an influence of tumor borders to the outcome of the patient with CRC ,where having an expansile smooth border goes with having a better outcome comparing to the irregular infiltrating tumor border. This is similar to what have been demonstrated by Koelzer and Lugli, 2014; for them, they also found an ameliorated outcome to patients with pushing border or expansile tumor growth pattern in contrast to those with infiltrating tumor border with 92.9% and 81.8% chance of a 5-year survival rate, respectively [21].

On a univariate analysis, patients with high immune response were found to live longer than those with low immune response with a *P* value = 0.001, patients without lympho-vascular invasion had a better outcome comparing with those with a positive LVI and with a *P* value = 0.018; however, only immune response came out as the sole factor independently associated with better survival on multiple logistic regression analysis (*P* value = 0.001, OR = 5.888, CI = (2.137–16.225)). This finding is similar to what have been found by Klintrup et al. [l] and Al-Sukhni et al. l [6,14]. As it has been documented, tumor microenvironment like immunodepression and angiogenesis play a major role in tumor progression, combining antiangiogenic therapy and immunotherapy seems to have the potential to tip the balance of the tumor microenvironment and improve the treatment response [22].

## 5. Conclusion

Among 101 colorectal resection specimens, patients presented with a wide age range 17–85 years with a mean of 54.26 and most of the patients were females, accounting to 53 (52.5%). Also, it has been shown that among 48 male patients presented, all 27 are below 50 years which is a big number of Rwandan males and it can be taken as a public health problem to deal with, with possible implementation of screening measures at the earliest. It has been shown that there is a considerable overall survival period of 46 months and with this 58% survived 2 years and 56% survived 3 years.

## Figures and Tables

**Table 1:** Distribution of age, sex, residence, main symptoms, duration of the symptoms, and biopsy site for the study participants.

Characteristics	*n*	Percent
*Age (n* *=* *101)*		
<50	42	41.6
51–75	54	53.5
>75	5	5

*Sex (n* *=* *101)*		
Male	48	47.5
Female	53	52.5

*Residence (n* *=* *101)*		
Eastern	17	16.8
Kigali City	33	32.7
Northern	13	12.9
Southern	25	24.8
Western	13	12.9

*Main symptom (n* *=* *101)*		
Abdominal pain	25	24.8
Constipation	1	1
Obstruction	19	18.8
Perforation	9	8.9
Rectal bleeding	47	46.5

*Duration of the symptoms (n* *=* *101)*		
< 6.months	52	51.5
> 24.months	4	4
13-24 months	7	6.9
7-12 months	38	37.6

*Anatomic site (n* *=* *101)*		
Ascending colon	28	27.7
Descending colon	27	26.7
Rectum	41	40.6
Transverse colon	5	5

*N* = number.

**Table 2:** Gross appearance and histopathological characteristics of colorectal carcinomas.

Characteristics	Numbers	Percent
*Gross appearance (n* *=* *101)*		
Infiltrating	69	68.3
Polypoid	32	31.7

*Histologic type (n* *=* *101)*		
Adenocarcinoma	61	60.4
Mucinous adenocarcinoma	40	39.6

*N* = number.

**Table 3:** Pathologic prognostic determinants of colorectal carcinomas.

Characteristics	Numbers	Percent
*Tumor grade (n* *=* *101)*		
Grade I	28	27.7
Grade II	55	54.5
Grade III	18	17.8

*Tumor stage (n* *=* *101)*		
Stage I	10	9.9
Stage II	21	20.8
Stage III	22	21.8
Stage IV	17	16.8
No stage	31	30.7

*Margin status (n* *=* *101)*		
Negative radial margin	72	71.3
Positive radial margin	29	28.7

*Lympho-vascular invasion (n* *=* *101)*		
Not present	52	51.5
Present	49	48.5

*Perineural invasion (n* *=* *101)*		
Not present	72	71.3
Present	29	28.7

*Tumor border (n* *=* *101)*		
Expansile	45	44.6
Irregular infiltrating	56	55.4

*Immune response (n* *=* *101)*		
High	72	71.3
Low	29	28.7

*N* = number.

**Table 4:** Correlation between sex and age group.

	Age groups		OR (95% CI)	*P*
	<50	>50		
Sex (*n* = 101)				
Male	26	22	2.7 (1.2–6.2)	0.01
Female	16	37		

*P* value by Fisher's exact test, *N* = number, OR : odds ratio, and CI : confidence interval.

**Table 5:** Correlation between prognostic determinants and tumor grade and stage.

Characteristics	Tumor grades		OR (95% CI)	*P* ^*∗*^	Tumor stages		OR (95% CI)	*P*
	Low	High			Low	High		
Immune response			(n = 101)				(n = 70)	
High	42	30	2.6 (1.1–6.5)	0.03	27	27	2.2 (0.7–7.2)	0.19
Low	10	19			5	11		
Lympho-vascular invasion			(n = 101)				(n = 70)	
Not present	38	14	6.7 (2.8–16.2)	<0.0001	19	21	1.18 (0.4–3.0)	0.7
Present	14	35			13	17		
Perineural invasion			(n = 101)				(n = 70)	
Not present	43	29	3.29 (1.3–8.24)	0.01	20	26	0.76 (0.3–2.0)	0.6
Present	9	20			12	12		
Tumor border			(n = 101)				(n = 70)	
Expansile	30	15	3.1 (1.4–7.0)	0.007	17	13	2.17 (0.8–5.7)	0.11
Irreg. infiltrating	22	34			15	25		
Margin status			(n = 101)				(n = 70)	
Neg. radial marg.	46	26	6,7 (2.4–18.7)	0.0002	23	28	0.91 (0.3–2.6)	0.8
Pos radial marg.	6	23			9	10		
Histologic type			(n = 101)				(n = 70)	
Carcinoma, NOS	40	21	4.4 (1.8–10.4)	0.0007	22	23	1.43 (0.5–3.8)	0.47
Carcinoma, mucinous	12	28			10	15		

^*∗*^ *P*value by Fisher's exact test; NOS: not otherwise specified; OR: odds ratio; and CI: confidence interval.

**Table 6:** The outcome of patients diagnosed with colorectal carcinoma operated on at CHUK.

Characteristics	Numbers	Percent
Deceased	36	35.6
Alive	56	55.4
Loss of follow-up	9	8.9
Total	101	100

**Table 7:** Logistic regression analysis of factors associated with survival in patients with colorectal cancer at CHUK.

Variables	Categories	*P* value	OR	95% C.I. for OR
Lympho-vascular invasion	Not present	0.333	0.647	[0.268–1.563]
Immune response	High	0.001	5.888	[2.137–16.225]

## Data Availability

The clinical and histological data used to support the findings of this study are available from the corresponding author upon request. The histological slides used to support the findings of this study may be released upon application to the CHUK Research Ethics Committee, who can be contacted at emmanuel.munyaneza@chuk.rw.
